# Steroid and metabolic hormonal profile of porcine serum *vis-à-vis* ovarian follicular fluid

**DOI:** 10.14202/vetworld.2016.1320-1323

**Published:** 2016-11-29

**Authors:** Soumen Naskar, S. Borah, Y. Vashi, R. Thomas, D. K. Sarma, J. Goswami, S. K. Dhara

**Affiliations:** 1 ICAR-National Research Centre on Pig, Guwahati - 781 131, Assam, India; 2 ICAR-Indian Institute of Agricultural Biotechnology, Ranchi - 834 010, Jharkhand, India; 3 Department of Physiology and Biochemistry, Lakhimpur College of Veterinary Science (AAU), Lakhimpur - 787 051, Assam, India; 4 Department of Veterinary Physiology, College of Veterinary Science (AAU), Guwahati - 781 022, Assam, India; 5 Division of Veterinary Biotechnology, ICAR-Indian Veterinary Research Institute, Izatnagar - 243 122, Uttar Pradesh, India

**Keywords:** cortisol, follicular fluid, pig, serum, T3, T4, testosterone

## Abstract

**Aim:**

This study was conducted to understand whether serum level of the steroid and metabolic hormones may be indicative of their level in ovarian follicular fluid (FF) in porcine, and its influence on fertility.

**Materials and Methods:**

Ovaries from pigs (n=32) of two genetic groups, namely, native (Ghungroo; n=16) and crossbred (Hampshire × Ghungroo; n=16) were collected. Both the genetic groups comprised gilts (n=8) and sows (n=8), and sows were in luteal phase of estrus cycle. FF was aspirated from small, medium and large follicles, and centrifuged for the collection of supernatant for further analysis. Blood samples were collected from the same animals, and serum was separated. Hormones, namely, cortisol, T3, T4 and testosterone were estimated by radioimmunoassay. Two-way ANOVA was used for analysis of data considering genetic background (native or crossbred), stage of reproductive life (gilt or sow), and source of sample (serum or FF) as fixed effects.

**Results:**

It was observed that all the hormones except cortisol differed significantly (p<0.01) based on genetic background. Stage of reproductive life and source of sample did not affect the studied hormonal level. Within the genetic groups, stage of reproductive life influenced T3 (p<0.01), cortisol (p<0.05) and testosterone (p<0.01) level in crossbred pigs as compared to T3 (p<0.01) only in native pigs. The level of T3 in serum, as well as FF, was higher (p<0.01) in Ghungroo gilts compared to sows. However, a reverse of this was observed in the case of crossbred pigs. The level of cortisol (p<0.05) and testosterone (p<0.01) was higher in crossbred sows than gilts in both serum and FF.

**Conclusion:**

The study revealed that serum level of the steroid and metabolic hormones is indicative of their level in the ovarian FF. Further, varying level of steroid and metabolic hormones in pigs based on genetic background may be due to variation in body size, rate of energy metabolism and stage of (re)productive life.

## Introduction

Ovarian follicular fluid (FF) is composed partly of secretions from the granulose cells and partly of exudates from plasma [1]. It provides a suitable microenvironment for the development, growth and maturation of the oocyte, and is vital for maintenance of fertility in mammals through its autocrine and paracrine regulation of physiological, biochemical and metabolic processes of the nuclear and cytoplasmic maturation of the oocyte and subsequent ovulation.

During follicular development, the theca cells produce androgens under the influence of follicle stimulating hormone (FSH), which diffuse to granulose cells where the androgens are transformed into estrogen under the influence of luteinizing hormone (LH) by the enzyme aromatase. The estrogen stimulates further growth and development of granulose cells and causes preovulatory surge of gonadotropins (LH) leading to ovulation. The steroid and metabolic hormonal composition of FF may influence steroidogenesis, oocyte maturation and quality, ovulation and transport of the oocyte to the oviduct as well as preparation of the follicle for subsequent corpus luteum formation and function [2].

Biochemical and metabolites concentration in the FF of bovine, sheep and human is widely reviewed [3], but corresponding information of porcine is not extensively studied. In the present experiment, hormonal profile namely cortisol, triiodothyronine (T3), thyroxine (T4) and testosterone in serum and FF of pigs of two different genetic groups was estimated for understanding their influence on sexual maturity and fertility.

## Materials and Methods

### Ethical approval

All animals including experimental animals were maintained under standard management condition with scientific feeding and health management practices. The animals were humanely treated, and the experiment was designed as per the guidelines of Committee for the Purpose of Control and Supervision of Experiment on Animals and Government of India guidelines and approved by Institutional Animal Ethics Committee. Humane slaughter of the animals was invariably practiced in R&D Pork Processing Plant of ICAR-NRC on Pig, Guwahati, an HACCP/ISO 9001:2008/FSSAI-certified facility.

### Sampling and radioimmunoassay

Ovaries from pigs (n=32) of two genetic groups, namely, native (Ghungroo; n=16) and crossbred (Hampshire × Ghungroo; n=16) were collected from R&D Pork Processing Plant. Both the genetic groups comprised gilts (n=8) and sows (n=8), and sows were in luteal phase of estrus cycle. The ovaries were kept in normal saline containing antibiotics (penicillin and streptomycin) for transport to the laboratory. FF was aspirated from small (5-9 mm), medium (6-10 mm) and large (>10 mm) follicles and centrifuged at 10,000 rpm for 15 min. Supernatant was collected and stored at −20°C for further analysis. Blood samples were collected from the same animals before their transport to the slaughter house; serum was separated and stored at −20°C for further analysis. Hormones namely cortisol, T3, T4 and testosterone were estimated by radioimmunoassay (RIA) using commercially available kits (Immunotech, A Beckman Coulter Company, France) as per manufacturer’s suggested protocol from the collected serum and FF.

### Statistical analysis

Two-way ANOVA [4] was used for analysis of data considering genetic background (native or crossbred), stage of reproductive life (gilt or sow) and sample source (serum or FF) as fixed effects.

## Results

The hormonal profiles of cortisol, T3, T4 and testosterone in serum and FF of native and crossbred pigs are presented in Table-1. It was observed that all the hormones except cortisol differed significantly (p<0.01) based on genetic background of pig (native or crossbred). Stage of reproductive life (gilt or sow) and sample source (serum or FF) did not affect the studied hormonal level across the genetic background.

**Table 1:** Metabolic and steroid hormone profile in serum and FF of two different genetic groups of pig.

Group	Source	Testosterone (ng/ml)		Cortisol (mmol/L)		T3 (nmol/L)		T4 (nmol/L)	
			
		Gilt	Sow	Gilt	Sow	Gilt	Sow	Gilt	Sow
G (n=16)	Serum	2.98^a^±0.78	2.74^a^±0.51	209.0±18.51	204.82±9.11	0.71^a^±0.01	0.32^a^±0.01	37.79^a^±0.98	31.28^a^±1.12
	FF	3.61^a^±0.54	3.04^a^±0.47	236.06±29.33	201.26±7.59	0.69^a^±0.04	0.30a±0.01	38.82^a^±6.05	32.14^a^±4.49
HS×G (n=16)	Serum	15.37^b^±3.91	23.47^b^±2.43	205.97±4.94	248.64±13.72	0.61^b^±0.04	0.86^b^±0.13	40.41^b^±2.26	42.81^b^±1.20
	FF	11.18^b^±3.21	26.34^b^±4.40	200.25±33.63	256.05±20.05	0.57^b^±0.08	0.90^b^±0.10	43.41^b^±3.70	40.45^b^±1.96

Means with different superscript within a column differ significantly (p<0.01). HS=Hampshire, G=Ghungroo, FF=Follicular fluid

**Figure-1:**
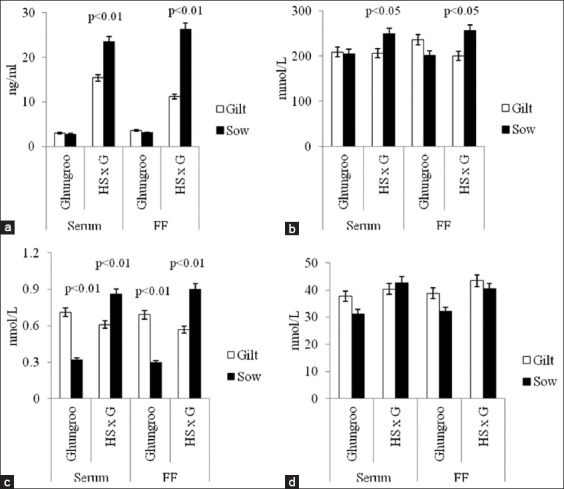
Comparison of metabolic and steroid hormones in serum and follicular fluid (FF) of different genetic groups of pigs based on stage of reproductive life (gilt and sow), (a) testosterone profile, (b) cortisol profile, (c) T3 profile, (d) T4 profile.

Within the genetic groups, stage of reproductive life influenced T3 (p<0.01), cortisol (p<0.05) and testosterone (p<0.01) level in crossbred pigs as compared to T3 (p<0.01) only in native pigs (Figure-1). The level of T3 in serum, as well as FF, was higher (p<0.01) in Ghungroo gilts compared to sows. However, a reverse of this was observed in the case of crossbred pigs. The level of cortisol (p<0.05) and testosterone (p<0.01) was higher in crossbred sows than gilts in both serum and FF.

## Discussion

A higher level of T3 in serum and FF of gilts might be due to higher metabolic requirement, and concurrent higher level of it in crossbred sows might be due to higher turn-over rate of folliculogenesis compared to native pigs. It may be interesting to note that the T3 level is reported to be higher in small follicles compared to large ones [5]. The T3 is reported to have a stimulatory effect on both FSH and LH to enhance steroid biosynthesis in porcine granulosa cells [6], whereas T4 has a stimulatory effect on FSH only [7]. Thus, T3 level *per se* exerts more influence to ovarian function than T4. However for achieving an optimum reproductive efficiency, both FSH and LH are required in optimum amount as FSH is responsible for follicular growth whereas LH is responsible for ovulation. There are limited and sometimes contradictory data available in the literature confirming the influence of thyroid hormones on ovarian function under *in vivo* conditions [8].

In a previous study conducted by Sheikh *et al*. [9], the negative correlation in testosterone concentration between serum and FF was observed, and lower level of testosterone in FF is reported to exert better influence on the fertilization process of the ova [10]. The higher level of testosterone in crossbred sows observed in the present experiment might be due to elevated level of T3 as it contributes positively to LH-induced androstenedione production by theca cells [11] and thyroid hormones directly alter granulose cell steroidogenesis in pigs [12] and humans [13].

In context of cortisol concentration in serum and FF, Lewicka *et al*. [14] observed concentrations of cortisol in FF that were approximately half than those concentrations in serum of human patients. Further, it was also evident that during any stress condition of the ovary like cystic follicles, the concentration was twice that of found in normal ovaries [15]. In the present experiment, it was observed that cortisol concentration did not differ significantly in different genetic groups. It might be due to normal cyclic ovarian condition as the ovarian follicles were thoroughly examined for excluding any pathological lesions, and FF was aspirated from healthy follicles. It is also evident that normally fluctuating concentrations of cortisol in FF of cattle play little or no active role in follicular differentiation *in vivo* [16]. In the present case, observed higher level of cortisol in serum and FF of crossbred sows might be due to enhanced steroidogenesis as cortisol is an intermediate in steroidogenesis pathway.

## Conclusions

The study revealed that serum level of the steroid and metabolic hormones is indicative of their level in FF in porcine. Further, the varying level of steroid and metabolic hormones in pigs based on genetic background may be due to variation in body size, rate of energy metabolism and stage of (re)productive life.

## Authors’ Contributions

SN and SKD planned and designed the experiment. Sampling was done by SB, YV and RT. RIA was carried out by JG. Data analysis and interpretation were done by SN, SB, YV and SKD. Manuscript was written by SB and YV, and edited by SN and DKS. All the authors read and approved the final manuscript.
